# Pregnancy outcomes of patients with retained products of conception following miscarriage treated with relugolix, an oral gonadotropin-releasing hormone antagonist

**DOI:** 10.3389/fmed.2026.1704529

**Published:** 2026-01-23

**Authors:** Satoko Sasatsu, Yosuke Ono, Dai Miyashita, Tatsuya Yoshihara, Kota Tanaka, Yuri Tada, So Owada, Akiko Nakagomi, Shoko Ikeda, Maki Ohgi, Eriko Ogasahara, Hikaru Tagaya, Yasuhiko Okuda, Osamu Yoshino

**Affiliations:** Department of Obstetrics and Gynecology, University of Yamanashi, Chuo City, Yamanashi, Japan

**Keywords:** assisted reproductive technology, GnRH antagonist, pregnancy outcome, relugolix, retained products of conception

## Abstract

**Background:**

Relugolix, an oral gonadotropin-releasing hormone (GnRH) antagonist, represents a potentially effective and less invasive therapeutic approach for retained products of conception (RPOC). However, its impact on subsequent pregnancy outcomes remains unclear. This study aimed to evaluate these outcomes following GnRH antagonist treatment for RPOC.

**Methods:**

This single-center cohort study encompassed 20 patients diagnosed with RPOC following miscarriage or abortion before 22 gestational weeks who were treated with oral relugolix, a GnRH antagonist, from January 2022 to July 2024. Following treatment completion and hysteroscopic confirmation of complete resolution, 12 patients subsequently conceived and were prospectively followed for pregnancy and neonatal outcomes. To contextualize outcomes, results were compared with the non-GnRH antagonist group managed at the same institution from 2014 to 2021 without GnRH antagonist exposure.

**Results:**

The GnRH antagonist group had fewer patients requiring surgical intervention than the non-GnRH antagonist group [30.0% (6/20) vs. 70.1% (54/77), *p* = 0.002], underscoring the reduced invasiveness of medical therapy. Of the 12 pregnancies following relugolix treatment, 4 led to early miscarriage and 8 progressed beyond 22 gestational weeks. The GnRH antagonist and non-GnRH antagonist groups exhibited comparable clinical pregnancy and live birth rates (53.3% vs. 73.3 and 33.3% vs. 48.9% per embryo transfer, respectively; not significant). Similarly, the interval from RPOC diagnosis in the previous pregnancy to gestational sac confirmation in the current pregnancy (328.9 ± 153.4 vs. 486.9 ± 658.8 days) and the interval from RPOC treatment completion to gestational sac confirmation (295.7 ± 166.6 vs. 445.8 ± 628.2 days) did not significantly differ between both groups.

**Conclusion:**

Relugolix therapy for RPOC was associated with preserved fertility and favorable pregnancy outcomes comparable to conventional management.

## 1 Introduction

**Figure 1:**
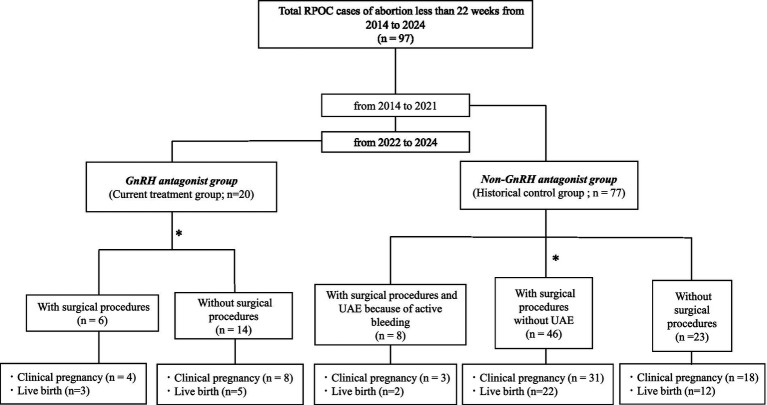
Study flow diagram of patients with retained products of conception (RPOC) following abortion before 22 gestational weeks between 2014 and 2024. A total of 97 patients were identified and divided into two cohorts: the GnRH antagonist group (current treatment group, treated between 2022 and 2024; *n* = 20) and the non-GnRH antagonist group (historical control group, treated between 2014 and 2021; *n* = 77). In the GnRH antagonist group, patients were initially managed with oral GnRH antagonist therapy. Surgical intervention was considered if there was no reduction in RPOC blood flow or lesion size within 4 weeks after treatment initiation, or in cases of active bleeding. Based on subsequent management, the GnRH antagonist group (current treatment group; *n* = 20) was classified into two subgroups: patients who required surgical procedures (*n* = 6) and those who were managed without surgical intervention (*n* = 14). In contrast, the non-GnRH antagonist group (historical control group; *n* = 77) was categorized into three subgroups according to treatment modality: patients who underwent surgical procedures with uterine artery embolization (UAE) because of active bleeding (*n* = 8), patients who underwent surgical procedures without UAE (*n* = 46), and patients who were managed without surgical intervention (*n* = 23). Clinical pregnancy and live-birth outcomes were evaluated separately for each subgroup.

Retained products of conception (RPOC) refer to residual gestational tissues that remain in the uterus following delivery, spontaneous miscarriage, or induced abortion (1–3). The incidence of RPOC is approximately 3% (4); it can cause post-pregnancy bleeding, which, in severe cases, may become life-threatening. With the increasing use of assisted reproductive technologies (ART), the incidence of placenta accreta has also risen, contributing to a higher risk of RPOC following abortion (5). Conventionally, expectant management—waiting for spontaneous expulsion—has been employed for RPOC. However, this strategy frequently involves extended bleeding and may take >3 months to completely resolve (2, 6). This prolonged waiting period can be emotionally and physically taxing for patients desiring to reconceive. Surgical interventions, including transcervical resection (TCR) or uterine evacuation, may be necessary when spontaneous expulsion does not occur (7–9). In cases of persistent or heavy bleeding, more invasive procedures, such as uterine artery embolization (UAE) or even hysterectomy, may be considered (10). These surgical options and UAE pose risks of complications, including intrauterine adhesions, reduced fertility, and increased miscarriage rates (11, 12), making them less favorable for patients desiring future pregnancies. To diminish the necessity for invasive surgical procedures, initially decreasing blood flow to the RPOC is crucial. Lowering vascularity reduces bleeding risk and facilitates a gentler surgical approach when intervention becomes necessary, minimizing endometrial damage (see Figure 1).

An oral gonadotropin-releasing hormone (GnRH) antagonist (relugolix) has been recently introduced for managing uterine fibroids (13). These agents reduce systemic estrogen levels without causing a flare-up effect, thereby reducing uterine blood flow. In our previous study, we demonstrated the efficacy of GnRH antagonists for RPOC following miscarriage. We observed that patients who received oral GnRH antagonist therapy demonstrated reduced vascularity and shrinkage in the size of the RPOC, and showed earlier menstrual recovery after RPOC diagnosis compared with patients managed without a GnRH antagonist (14). However, the pregnancy outcomes following GnRH antagonist treatment for RPOC remain unclear. We aimed to prospectively investigate the reproductive outcomes of patients with RPOC treated with oral GnRH antagonists.

## 2 Methods

### 2.1 Study design and setting

This single-center cohort study evaluated reproductive and perinatal outcomes following medical management of RPOC with an oral GnRH antagonist at the University of Yamanashi Hospital. This study combined a prospectively assembled case series of consecutively treated patients with a comparison cohort of non-GnRH antagonist–treated patients who were managed at the same institution prior to the introduction of relugolix for RPOC. The non-GnRH antagonist cohort was derived from our previously reported retrospective study of RPOC management at this center. The present cohort comprised the same 20 and 77 patients in the GnRH antagonist and non-GnRH antagonist groups, respectively, described in our previous study (14); in this follow-up study, we extended the analysis to evaluate their subsequent reproductive and perinatal outcomes.

The Ethics Committee of the University of Yamanashi Hospital approved this study (approval number: 2761). All participants provided written informed consent before inclusion. This study was conducted in accordance with the Declaration of Helsinki.

### 2.2 Participants

Between January 2022 and July 2024, 20 patients with RPOC following spontaneous or induced abortion at <22 gestational weeks received relugolix (40 mg orally, once daily in the evening). RPOC was diagnosed by transvaginal ultrasonography with color Doppler, confirming intrauterine echogenic material with demonstrable blood flow. Intralesional vascularity was assessed using the Gutenberg classification, a color Doppler–based grading system ranging from grade 0 (no detectable blood flow) to grade III (marked hypervascularity), as previously described (14, 15). Briefly, Gutenberg grade I indicates minimal intralesional vascularity with sparse color Doppler signals; grade II represents moderate vascularity with clearly detectable but non-dominant blood flow within the lesion; and grade III denotes marked hypervascularity characterized by abundant color Doppler signals, often showing high-velocity, low-resistance flow patterns suggestive of arteriovenous shunting (15). Only cases with Doppler-detectable intralesional vascularity corresponding to Gutenberg classification grade I–III were considered eligible for inclusion, to ensure clinical homogeneity with respect to lesion activity.

Of the 20 treated patients, 12 subsequently achieved pregnancy and were included in the outcome analyses. The exclusion criteria for the relugolix cohort were gestational age ≥22 weeks at the index abortion, molar pregnancy or malignancy, significant systemic disease precluding follow-up, or incomplete clinical records. All eligible patients meeting the inclusion criteria during the study period were consecutively enrolled, irrespective of treatment response, to minimize selection bias.

To contextualize outcomes, we assembled a non-GnRH antagonist comparison group consisting of patients managed at our institution between January 2014 and December 2021, during a period when surgical indications and RPOC management protocols had become standardized. These patients were identified using the same eligibility criteria and diagnostic approach as the relugolix-treated cohort and received conventional management, including expectant management and/or surgical treatment (transcervical resection or dilation and curettage), without exposure to GnRH antagonists.

Cases without Doppler-detectable vascularity (Gutenberg grade 0) were excluded from both cohorts to ensure comparability with respect to lesion activity and bleeding risk.

For the present study, we analyzed patients in the GnRH antagonist group who subsequently conceived following RPOC resolution. Relugolix was initiated following the diagnosis of RPOC and, in principle, administered for 2–4 weeks, with the exact duration determined based on Doppler findings and clinical judgment. Serial ultrasonography and Doppler reassessments were performed at approximately 2-week intervals. Treatment was generally discontinued after Doppler flow resolution; however, in selected cases, administration was extended beyond the standard period at the discretion of the treating physician. Surgical intervention was considered for persistent flow/size of approximately >4 weeks or for active bleeding, consistent with institutional criteria (14). Following completion of relugolix treatment and resumption of menstruation, all patients underwent hysteroscopic evaluation to confirm complete resolution of RPOC, after which permission to attempt conception was granted.

Regarding endometrial thickness, measurements were obtained at the time of RPOC diagnosis and at the time pregnancy was permitted in both the GnRH antagonist and non-GnRH antagonist groups, representing clinically corresponding time points for group comparison. Permission to attempt conception was determined by the attending physician at a follow-up visit after the resumption of menstruation following complete resolution of RPOC. In addition, in the GnRH antagonist group, endometrial thickness was also assessed at the time of RPOC resolution following treatment to evaluate treatment-related changes. In contrast, in the non-GnRH antagonist group, RPOC resolution typically occurred via spontaneous expulsion or surgical intervention, and therefore a comparable intermediate post-treatment assessment time point was not available.

### 2.3 Outcomes and follow-up

The following were the primary reproductive outcomes: achievement of clinical pregnancy (intrauterine gestational sac), ongoing pregnancy, and live birth in the first pregnancy attained following RPOC resolution. Secondary perinatal outcomes encompassed the following: gestational age at delivery, mode of delivery, birth weight, and obstetric complications (e.g., postpartum hemorrhage, placenta previa or placenta accreta spectrum, and need for blood transfusion or interventional radiology). When relevant, we recorded whether conception occurred naturally or via ART. Patients were followed from RPOC treatment completion to pregnancy outcome (delivery or pregnancy loss) or last available clinical contact within the study period. For the GnRH antagonist group, outcome data were obtained from institutional medical records using the same definitions and time-anchoring to the first conception following RPOC resolution.

### 2.4 Statistical analysis

Continuous variables were presented as means ± standard deviations or medians (ranges) and compared using Student’s *t*-test or the Mann–Whitney *U* test, as appropriate. Categorical variables were compared using Fisher’s exact test. Two-sided *p* < 0.05 was considered statistically significant. Statistical analyses were performed using JMP (SAS Institute, Cary, NC, USA), consistent with our previous RPOC study. Prior to selecting parametric or non-parametric tests, the normality of continuous variables was assessed using the Shapiro–Wilk test. Based on the results of this test, appropriate statistical methods were chosen, and the specific tests applied are indicated in the footnotes of each table.

To account for potential confounding effects of baseline clinical characteristics on fertility outcomes, we additionally performed propensity score–based adjustment. Because the number of patients who achieved pregnancy after RPOC treatment was limited, we considered that constructing a multivariable regression model including multiple covariates could result in model overfitting and reduced statistical robustness. Therefore, we selected propensity score–based matching as a more appropriate method to address confounding while preserving interpretability. Propensity scores were estimated using prespecified clinically relevant covariates, including age, use of ART, RPOC size, vascularity grade of RPOC, history of previous uterine surgical procedures, and the presence of endometriosis. Nearest-neighbor propensity score matching was conducted to improve comparability between the GnRH antagonist and non-GnRH antagonist groups. A 1:3 matching ratio was applied, matching 20 patients in the GnRH antagonist group to 60 patients selected from the original non-GnRH antagonist cohort (*n* = 77). Covariate balance before and after matching was assessed using standardized mean differences (SMDs), with values <0.1 considered indicative of adequate balance. Propensity score estimation, matching procedures, and balance diagnostics were performed using Python (Python Software Foundation, https://www.python.org), with relevant statistical and data analysis libraries. The results of these analyses are presented in Supplementary Table 1, Supplementary Figure 1.

To evaluate the potential impact of temporal bias related to differences in treatment era, we additionally performed sensitivity analyses restricted to cases treated from 2020 onward, during a period when clinical practice and management strategies for RPOC at our institution had become more consistent. The results of these analyses are presented in the Supplementary Table 2.

## 3 Results

### 3.1 Baseline characteristics and treatment details

Prior to the main analyses, propensity score matching was performed as a supplementary analysis to evaluate the potential impact of baseline confounding (Supplementary Table 1a). The results showed that key outcomes, including pregnancy and live birth rates, were comparable before and after matching, with no material differences in the interpretation of the findings (Supplementary Table 1b). Therefore, the primary analyses in this study were conducted using the full, unmatched cohort (GnRH antagonist group, *n* = 20; non-GnRH antagonist group, *n* = 77) to preserve sample size and reflect real-world clinical practice. No differences in age, body mass index, gravidity, parity, and the maximum diameter of RPOC at diagnosis were observed between the two groups (Table 1). The proportion of patients requiring surgical intervention for RPOC differed significantly between the two groups: 6 of 20 patients (30.0%) in the GnRH antagonist group versus 54 of 77 patients (70.1%) in the non-GnRH antagonist group (*p* = 0.002), indicating that fewer surgical procedures were required in the GnRH antagonist group. The median duration of relugolix administration before pregnancy was 28 (range, 8–57) days.

**Table 1:** Clinical backgrounds of the GnRH antagonist and non-GnRH antagonist groups.

RPOC cases (*n* = 97)	GnRH antagonist (*n* = 20)	Non-GnRH antagonist (*n* = 77)	*p* value
Age (years)	34.0 ± 6.2	33.8 ± 5.8	0.89^a^
Body mass index (kg/m^2^)	22.0 ± 4.3	21.1 ± 3.0	0.39^a^
Gravidity	1.9 ± 1.2	2.0 ± 1.4	0.75^b^
Parity	0.7 ± 1.0	0.7 ± 1.0	0.91^b^
Assisted reproductive technology	12 (60.0%)	35 (45.5%)	0.364^c^
Spontaneous abortion	16 (80%)	66 (85.7%)	0.777^c^
Artificial abortion	4 (20%)	11 (14.3%)	0.777^c^
Surgery for abortion	7 (35%)	26 (33.8%)	0.872^c^
History of dilation and curettage	13 (65%)	30 (39%)	0.045^c^
History of hysteroscopic transcervical resection	2 (10%)	2 (2.6%)	0.187^c^
History of cesarean section	3 (15%)	2 (2.6%)	0.058^c^
Ovarian endometrioma	1 (5%)	3 (3.9%)	1.000^c^
Adenomyosis	2 (10%)	1 (1.3%)	0.107^c^
Gestational age at diagnosis of abortion	9.0 (8.0–10.3)	16.5 (6–21)	0.85^b^
Maximum diameter of RPOC at diagnosis (mm)	26.5 (12–59)	57.5 (5–78)	0.59^b^
Surgical procedures for RPOC	6 (30%)	54 (70.1%)	0.002^c^
Hysteroscopic transcervical resection	6 (30%)	33 (42.9%)	0.240^c^
Dilation and curettage	0	9 (11.7%)	0.110^c^
Uterine artery embolism	0	8 (10.4%)	0.170^c^
The duration of relugolix administration (days)	28 (8–57)	–	NA
Endometrial thickness at RPOC diagnosis (mm)	9.2 (7.2–10.0)	10 (7.9–11.6)	0.361^b^
Endometrial thickness at completion of treatment (mm)	4.6 (4.2–7.3)	–	NA
Endometrial thickness at permission for pregnancy (mm)	8.9 (7.1–10.4)	7.8 (6.2–9.1)	0.156^b^
Vascularity grade of RPOC at diagnosis (Gutenberg classification)	II (I–III)	I (I–III)	0.102^b^
Vascularity grade 1 month after RPOC diagnosis (Gutenberg classification)	0 (0–I)	I (0–III)	<0.0001^b^
Change in vascularity grade from diagnosis to follow-up (baseline-post grade of Gutenberg classification)	2 (1–3)	0 (0–2)	<0.001^b^

Data are presented as medians (ranges) or *n* (%). *p*-Values <0.05 are considered statistically significant. Student’s *t*-test or Mann–Whitney *U* test was used for continuous variables, and Chi-square test or Fisher’s exact test was used for categorical variables, as appropriate. The specific test used for each variable is indicated in the table. RPOC; retained products of conception, GnRH; gonadotropin-releasing hormone, NA; not applicable.

a Student’s *t*-test.

b Mann–Whitney *U* test.

c Fisher’s exact test.

### 3.2 Recovery after RPOC treatment and time to subsequent pregnancy

In a comparison of endometrial thickness between the GnRH antagonist group and the non-GnRH antagonist group, the endometrial thickness at the time of RPOC diagnosis in the GnRH antagonist group was 9.2 (7.2–10.0) mm. In this group, endometrial thickness transiently decreased to 4.6 (4.2–7.3) mm at the time of RPOC resolution following treatment and subsequently recovered to 8.9 (7.1–10.4) mm at the time pregnancy was permitted.

In contrast, in the non-GnRH antagonist group, the corresponding endometrial thicknesses at the time of RPOC diagnosis and at the time pregnancy was permitted were 10.0 (7.9–11.6) mm and 7.8 (6.2–9.1) mm, respectively (Table 1). When comparisons were restricted to these clinically corresponding time points, no significant differences in endometrial thickness changes were observed between the two groups. These findings indicate that although GnRH antagonist treatment was associated with a transient reduction in endometrial thickness during treatment, endometrial recovery was preserved by the time pregnancy was permitted.

With respect to RPOC vascularity grade, there was no significant difference between the GnRH antagonist and non-GnRH antagonist groups at the time of RPOC diagnosis. In the GnRH antagonist group, the vascularity grade significantly decreased at the first standardized post-treatment evaluation, which was scheduled approximately 1 month after treatment initiation, improving from a median of II (range, I–III) at diagnosis to 0 (0–I) (*p* < 0.0001). Ultrasound assessments were performed according to a predefined follow-up protocol, and in cases where vascularity disappeared earlier, treatment was discontinued and the vascularity grade at the time of treatment discontinuation was used for analysis.

In the non-GnRH antagonist group, vascularity grade also showed a statistically significant reduction (*p* = 0.012), with the median value changing from I (I–III) at diagnosis to I (0–III) at the same standardized post-treatment evaluation time point. However, the magnitude of vascularity reduction—defined as the difference between baseline and follow-up grades (baseline − follow-up)—was significantly greater in the GnRH antagonist group than in the non-GnRH antagonist group [II (I–III) vs. 0 (0–II), *p* < 0.001], indicating a more pronounced reduction in RPOC vascularity associated with GnRH antagonist treatment (Table 1).

### 3.3 Pregnancy outcomes after RPOC treatment

Following relugolix treatment for RPOC, 12 pregnancies occurred, 9 of which were achieved through ART and 4 led to early miscarriage. In the GnRH antagonist group, the clinical pregnancy rate per embryo transfer was 53.3%, and the live-birth rate per embryo transfer was 33.3%. In the non-GnRH antagonist group, the corresponding rates were 73.3 and 48.9%, respectively, with no significant differences noted between both groups (Table 2). When natural pregnancies were also included, the clinical pregnancy rate and live birth rate per patient did not differ between the two groups.

**Table 2:** Assisted reproductive technology and overall pregnancy outcomes in the GnRH antagonist and non-GnRH antagonist groups.

RPOC cases (*n* = 97)	GnRH antagonist (*n* = 20)	Non-GnRH antagonist (*n* = 77)	*p* Value
Assisted reproductive technology outcome			
Number of embryo transfer (cycles)	15	45	0.271
Clinical pregnancy	8 (53.3%)	33 (73.3%)	0.262
Live birth	5 (33.3%)	25 (48.9%)	0.233
Miscarriage rate/per clinical pregnancy	3 (37.5%)	11 (33.3%)	1
Overall pregnancy outcome			
Clinical pregnancy	12 (60%)	52 (67.5%)	0.712
Live birth	8 (40%)	36 (46.8%)	0.773

Data are presented as medians (ranges) or *n* (%). *p*-Values <0.05 are considered statistically significant. RPOC, retained products of conception; GnRH, gonadotropin-releasing hormone.

Statistical comparisons between the two groups were performed using Fisher’s exact test.

### 3.4 Obstetric and neonatal outcomes

The interval from RPOC diagnosis in the previous pregnancy to confirmation of a gestational sac in the subsequent pregnancy was 328.9 ± 153.4 days in the GnRH antagonist group and 486.9 ± 658.8 days in the non-GnRH antagonist group. Similarly, the interval from completion of RPOC treatment to confirmation of a gestational sac was 295.7 ± 166.6 days and 445.8 ± 628.2 days in the GnRH antagonist and non-GnRH antagonist groups, respectively. Neither interval differed significantly between the two groups (Table 3). These data were restricted to patients who subsequently achieved pregnancy and was not designed to assess whether GnRH antagonist therapy shortens the duration of RPOC treatment itself. Nevertheless, the lack of a significant difference in these time intervals suggests that GnRH antagonist therapy is unlikely to have a negative impact on the timing of subsequent conception. Among the eight pregnancies that progressed beyond 22 gestational weeks, three developed obstetric complications: one case of gestational diabetes mellitus requiring insulin treatment at 27 weeks, one case of gestational hypertension followed by proteinuria and preeclampsia at 38 weeks, and one case of genital bleeding due to a cervical polyp excised at 12 weeks. No cases of preterm delivery occurred, and the frequency of obstetric complications did not differ between the two groups (Tables 3, 4). Three patients underwent cesarean section, one had a forceps-assisted delivery, and four delivered vaginally without intervention. Indications for cesarean section included a history of uterine myomectomy in two patients and a previous cesarean section in one. The forceps-assisted delivery was performed because of fetal heart rate deceleration. No emergency cesarean sections were required due to fetal distress or labor arrest (Table 4). No significant differences were observed between the two groups with respect to gestational age at delivery, cesarean section rate, blood loss at delivery, neonatal intensive care unit (NICU) admission rate, or postpartum RPOC incidence. One neonate experienced complications related to large-for-gestational-age status; however, no congenital anomalies, respiratory disorders, hypoglycemia, or NICU admissions were reported (Table 3).

**Table 3:** Obstetric complications and delivery outcomes in pregnancies of the GnRH antagonist and non-GnRH antagonist groups.

Obstetric complications and delivery outcomes	GnRH antagonist (*n* = 8, total number of pregnancies: 12)	Non-GnRH antagonist (*n* = 36, total number of pregnancies: 52)	*p* value
Interval from diagnosis of RPOC in the previous pregnancy to the confirmation of gestational sac in the current pregnancy (days)	328.9 ± 153.4	486.9 ± 658.8	0.458^a^
Interval from completion of RPOC treatment to confirmation of gestational sac (days)	295.7 ± 166.6	445.8 ± 628.2	0.461^a^
The maximum diameter of RPOC (mm)	19.5 (12–50)	22.5 (5–60)	0.599^b^
Miscarriage	4 (33.3%)	16 (30.8%)	0.713^c^
Preterm delivery	0	3 (8.6%)	1.000^c^
Placenta previa	0	1 (2.6%)	1.000^c^
Fetal growth restriction	1 (8.3%)	3 (8.6%)	1.000^c^
Hypertensive disorder of pregnancy	1 (8.3%)	2 (5.7%)	0.539^c^
Preterm rupture of membrane	0	4 (11.4%)	0.561^c^
Gestational weeks	38.9 (37.0–41.4)	38.8 (31.4–41.6)	0.897^b^
Cesarean section	3 (25.0%)	22 (42.3%)	0.338^c^
Blood loss at delivery (mL)	675.8 (362–1,186)	917.2 (217–1977)	0.197^b^
NICU admission	0 (0%)	4 (11.4%)	0.561^c^
Recurrence of RPOC	1 (8.3%)	3 (6.4%)	0.306^c^

Data are presented as means ± standard deviations or as *n* (%).

*p*-Values <0.05 are considered statistically significant. GnRH, gonadotropin-releasing hormone.

Student’s *t*-test or Mann–Whitney *U* test was used for continuous variables, and Chi-square test or Fisher’s exact test was used for categorical variables, as appropriate.

The specific test used for each variable is indicated in the table.

a Student’s *t*-test.

b Mann–Whitney *U* test.

c Fisher’s exact test.

**Table 4:** Clinical courses of pregnant cases in GnRH antagonist group (*n* = 12).

Case	Maternal age (years)	Diameter of RPOC (mm)	Duration of taking GnRH antagonist (days)	Mode of pregnancy	Obstetric outcomes	GW at delivery	Mode of delivery	Indications for expedited delivery	Birth weight (g)	Blood loss at delivery (mL)	Neonatal outcome	NICU admission	Apgar score at 1 min (points)	Apgar score at 5 min (points)	UApH	Recurrence of RPOC
1	35	12	25	ART	Normal course	37	VD	–	2,746	398	Normal	No	8	10	7.37	No
2	38	35	28	ART	Normal course	38.6	CS	History of CS	2,914	801	Normal	No	8	9	7.31	Yes
3	39	23	28	ART	GDM	38.4	CS	History of myomectomy	2,794	711	Normal	No	8	9	7.29	No
4	40	50	28	ART	Normal course	38.9	CS	History of myomectomy	2,962	1,186	Normal	No	8	9	7.19	No
5	38	15	29	Spontaneous	Normal course	40	Forceps assisted delivery	NRFS	3,192	743	Normal	No	8	10	7.18	No
6	37	19	14	ART	GH, PE	38.7	VD	–	3,132	673	Normal	No	8	9	7.31	No
7	28	15	39	ART	Normal course	39	VD	–	3,430	362	LGA	No	8	9	7.35	No
8	31	20	57	Spontaneous	Bleeding from cervical polyp and removal	41.6	VD	–	3,138	533	Normal	No	8	9	7.32	No
9	33	20	14	ART	Spontaneous abortion (7 GW)	–	–	–	–	–	–	–	–	–	–	No
10	41	19	14	ART	Spontaneous abortion (9 GW)	–	–	–	–	–	–	–	–	–	–	No
11	25	23	66	Spontaneous	Spontaneous abortion (7 GW)	–	–	–	–	–	–	–	–	–	–	No
12	39	15	29	ART	Spontaneous abortion (9 GW)	–	–	–	–	–	–	–	–	–	–	No

GnRH, Gonadotropin-Releasing Hormone; ART, assisted reproductive technology; VD, vaginal delivery; CS, cesarean section; NRFS, non-reassuring fetal status; LGA, large for gestational age.

The details of 12 pregnant cases in GnRH antagonist group are shown in Table 4.

### 3.5 Postpartum course in a representative case

In one patient with a history of uterine myomectomy, the placenta was attached to the right fundal wall, and manual placental removal was required at delivery. At 1 month postpartum, a 23-mm RPOC was detected at the same site. Ultrasonography demonstrated no vascularity, and the patient was breastfeeding; therefore, conservative management without GnRH antagonist therapy was chosen. Over time, the retained tissue gradually decreased in size.

### 3.6 Sensitivity analyses addressing potential temporal bias

To assess the potential impact of temporal bias, we conducted supplemental sensitivity analyses restricted to pregnancies achieved in cases treated from 2020 onward (Supplementary Table 2). In this restricted cohort, baseline clinical characteristics were comparable between the GnRH antagonist and non-GnRH antagonist groups. Overall pregnancy outcomes, including clinical pregnancy rate, live birth rate, miscarriage rate, gestational age at delivery, obstetric complications, and neonatal outcomes, did not differ significantly between groups. Although the clinical pregnancy rate per embryo transfer was higher in the non-GnRH antagonist group, this difference did not translate into significant differences in overall live birth or obstetric outcomes. These findings were consistent with the primary analysis and suggest that the observed results are unlikely to be driven solely by temporal changes in clinical practice.

## 4 Discussion

To our knowledge, this study is the first to evaluate pregnancy outcomes following oral treatment with a GnRH antagonist for RPOC. In the present study, pregnancy, obstetric, and live-birth outcomes following relugolix therapy were comparable to those observed with conventional management, suggesting preserved fertility after treatment.

Given the retrospective nature of the study and the known influence of baseline fertility-related characteristics on reproductive outcomes, we carefully assessed the comparability of the treatment groups using the same data set as in our previous report (14). To address potential confounding due to differences in patient background, we performed propensity score matching using clinically relevant covariates, including age, use of ART, RPOC size and vascularity grade, prior uterine surgical procedures, and comorbid endometriosis. After matching, covariate balance between the relugolix and non-relugolix groups was adequately achieved, and importantly, pregnancy and live-birth outcomes remained consistent before and after matching. These findings suggest that, although residual bias cannot be completely excluded, the comparison between the two groups is acceptable and that the observed outcomes are unlikely to be primarily driven by imbalances in measured baseline characteristics.

Originally, GnRH antagonists were introduced as oral agents for uterine fibroids; unlike GnRH agonists, they do not cause a flare-up effect (16), making them more clinically convenient. Accumulating evidence has recently demonstrated their efficacy across various gynecological indications. In the context of pretreatment for myomectomy, GnRH antagonists have been demonstrated to decrease fibroid volume, improve hemoglobin levels, reduce the need for transfusion, and increase the feasibility of minimally invasive approaches, including laparoscopic surgery instead of laparotomy (17). Moreover, their use has expanded into the field of infertility treatment, further broadening their application in obstetrics and gynecology (18). In RPOC management, Goda et al. (19) first reported the successful use of a GnRH antagonist for a placental polyp associated with uterine arteriovenous malformation.

Previously, we reported that relugolix short-term administration for RPOC was associated with reduced vascularity and lesion size, potentially facilitating spontaneous expulsion and minimizing the need for surgical intervention (14). Although the precise pathophysiological mechanisms underlying RPOC have not been fully elucidated, accumulating evidence suggests that RPOC represents not merely passive residual tissue but a vascularized intrauterine lesion sustained by persistent perfusion. Normally, placental separation is followed by closure of spiral arteries, apoptosis of trophoblasts, and physiological uterine involution. In contrast, RPOC is thought to arise when these processes are incomplete, allowing biologically active trophoblastic tissue—particularly intermediate trophoblasts—to persist and maintain blood supply through the production of angiogenic factors (20). Consistent with this concept, RPOC frequently demonstrates prominent color Doppler flow characterized by high-velocity, low-resistance, and arteriovenous malformation–like waveforms, indicating pathological angiogenesis and sustained uterine perfusion (15, 21). Such hypervascular RPOC lesions have been reported to be less likely to resolve spontaneously and to carry a higher risk of prolonged bleeding. Therefore, reduction of vascularity appears to be a key mechanism underlying lesion regression. In the present study, administration of a GnRH antagonist resulted in a progressive decrease in blood flow velocity within the retained tissue, followed by disappearance of Doppler-detectable flow and eventual resolution of the lesion. Estrogen is known to enhance uterine arterial blood flow through nitric oxide–mediated vasodilation, whereas suppression of estrogen increases uterine vascular resistance and reduces perfusion (22). By rapidly inhibiting pituitary GnRH receptors and suppressing luteinizing hormone and follicle-stimulating hormone secretion without a flare-up phenomenon, GnRH antagonists induce a hypoestrogenic state that may attenuate angiogenic signaling and interrupt blood supply to vascularized RPOC. Taken together, our observed reduction and eventual disappearance of RPOC perfusion provides a plausible mechanistic explanation for lesion regression with relugolix therapy, and this vascular regression likely facilitates detachment and spontaneous expulsion of retained placental tissue, while simultaneously minimizing the need for invasive surgical intervention and preserving endometrial integrity, thereby providing a plausible biological link between reduced RPOC vascularity and favorable subsequent fertility outcomes.

Nevertheless, data on the impact of GnRH antagonist therapy on subsequent fertility remain scarce. However, a recent report has indicated that when used for managing endometriosis-related pain, even after long-term administration of up to 24 months, most patients resumed menstruation within 2 months of discontinuation (23). In our previous study involving 20 patients with RPOC, the interval from RPOC resolution to the first post-treatment menses was 18.3 ± 17.9 days, significantly shorter than that with conventional management (14). These observations support that the pharmacological reversibility of GnRH antagonists is compatible with timely resumption of fertility treatment in the setting of RPOC.

Although RPOC can spontaneously resolve in some cases, others may be refractory to expectant management or prove challenging to treat even with surgery. For patients desiring future pregnancy, prolonged persistence of RPOC may impose a substantial psychological burden.

In our previous study, patients treated with a GnRH antagonist demonstrated a significantly shorter interval from RPOC diagnosis to menstrual resumption compared with those managed without a GnRH antagonist [median 14.5 (9–71) days vs. 26.0 (6–95) days, *p* = 0.002] (14).

Earlier recovery of menstruation following successful conservative management may therefore help reduce psychological stress and facilitate earlier resumption of fertility treatments. In addition, avoidance of surgical procedures may further reduce physical and emotional burden. Importantly, the comparable pregnancy and delivery outcomes observed in the present study indicate that this less invasive approach does not compromise subsequent reproductive potential.

Furthermore, avoiding surgical procedures may reduce physical and emotional burdens on the patient. Importantly, our finding of comparable pregnancy and delivery outcomes suggests that this less invasive approach does not appear to compromise subsequent reproductive potential.

The pregnancy, obstetric complication, and live-birth rates following relugolix therapy being comparable to those with conventional management, indicating preserved fertility, represent a notable finding of this study. Furthermore, although the groups showed no significant differences in terms of the interval from diagnosis or RPOC treatment completion to subsequent conception, the lower proportion of surgical interventions in the relugolix group may represent an additional advantage. As surgical procedures are associated with psychological stress, physical invasiveness, and the risk of intrauterine adhesions, achieving resolution with medical therapy alone may be particularly beneficial for patients desiring future fertility. Additionally, avoiding surgical interventions positively impacts patients’ quality of life.

We here observed one case of postpartum recurrence of RPOC; however, its frequency was lower than the recurrence rates following surgical treatment reported in the literature (24). This case had previously undergone TCR for RPOC following miscarriage, subsequently conceived through ART, and required manual placental removal at delivery. Noguchi et al. reported an association between placental forceps use in 98 cases of RPOC following miscarriage and an increased risk of recurrence (3); in our case, manual placental removal may have been a contributing factor. Further clarification of recurrence risk factors and the development of preventive strategies are crucial for optimizing RPOC management.

This study had several limitations. Although our results suggest that fertility can be preserved after treatment with an oral GnRH antagonist relugolix for RPOC, the limited number of subsequent pregnancies represents a major limitation and restricts the statistical power to detect differences in fertility and pregnancy outcomes. In addition, the comparison group consisted of a non-GnRH antagonist cohort managed at our institution prior to the introduction of relugolix. Although this cohort was restricted to cases treated after 2014, when surgical standards and management strategies for RPOC had become more consistent, residual bias inherent in the use of the non-GnRH antagonist group as a historical control cannot be completely excluded. Differences in clinical practice over time, patient characteristics, and unmeasured confounders may have influenced the results. While propensity score matching improved balance in measured baseline characteristics and supported the comparability of the groups, residual confounding due to unmeasured or incompletely captured factors remains possible, as in any observational study.

To further address concerns regarding potential temporal bias, we conducted supplemental sensitivity analyses restricted to cases treated from 2020 onward, a period during which clinical practice, surgical indications, imaging assessment, and ART protocols at our institution had become more uniform. These analyses yielded results consistent with those of the primary analysis, supporting the robustness of our findings; however, they do not fully eliminate the possibility of era-related bias.

In conclusion, this study suggests that conservative management of RPOC with the oral GnRH antagonist relugolix may preserve subsequent fertility and allow for favorable pregnancy and obstetric outcomes comparable to conventional management. While the findings should be interpreted with caution due to the exploratory nature of the study and limited sample size, they provide clinically relevant preliminary evidence supporting the reproductive safety of GnRH antagonist therapy for RPOC. Larger prospective multicenter studies are warranted to confirm these observations and to further define the role of GnRH antagonists in fertility-preserving RPOC management.

## Data Availability

The original contributions presented in the study are included in the article/Supplementary material, further inquiries can be directed to the corresponding authors.
