# Supplementary material for: Pregnancy outcomes of patients with retained products of conception following miscarriage treated with relugolix, an oral gonadotropin-releasing hormone antagonist
Source: Front Med (Lausanne). 2026 Jan 23;13:1704529. doi: 10.3389/fmed.2026.1704529 (PMC12876250; doi:10.3389/fmed.2026.1704529)
Supplement: Supplementary file 3 [file Data_Sheet_1.pdf]

Supplementary Figure 1.

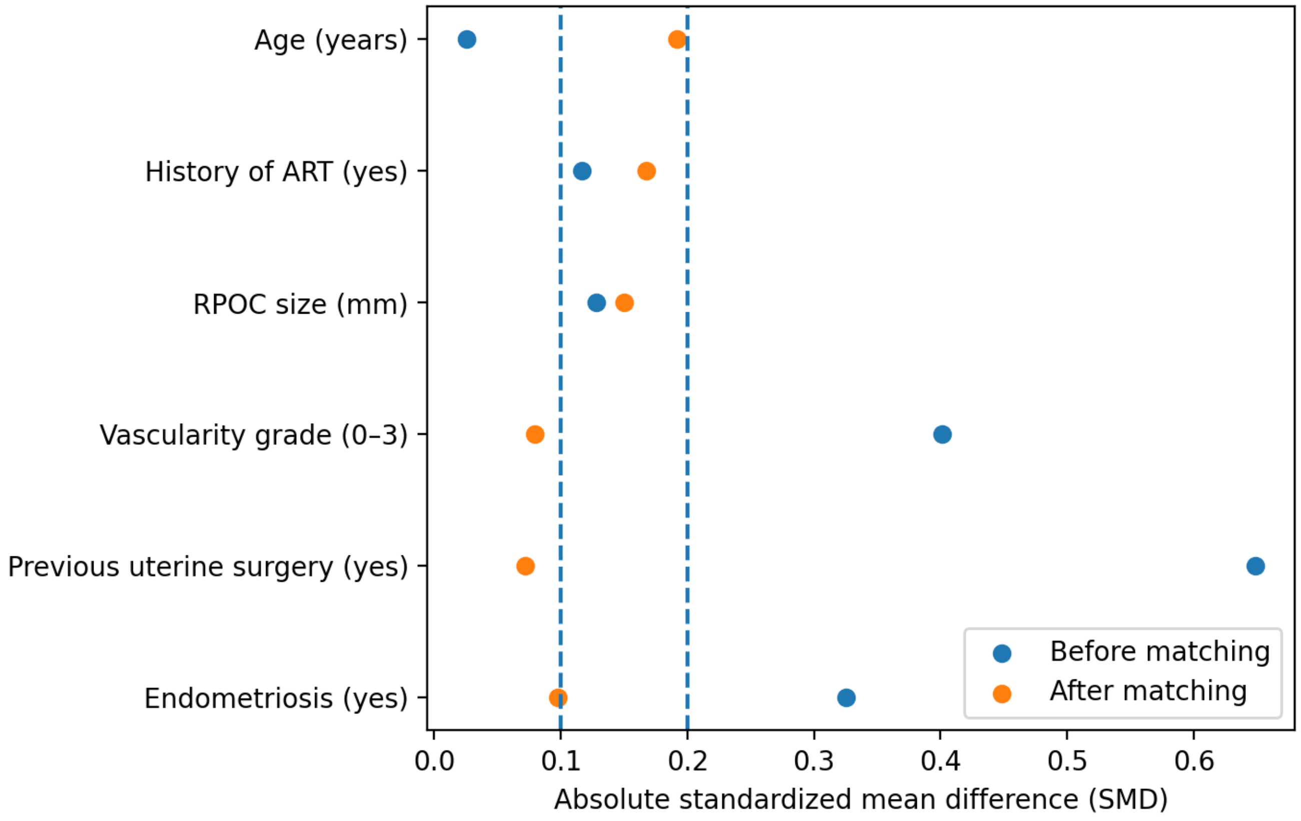

Supplementary Figure 2. Love plot of covariate balance before and after propensity score matching
Dots represent the absolute standardized mean differences (|SMD|) for each covariate before matching (blue) and after matching (orange). Vertical dashed lines indicate commonly used thresholds for acceptable balance (|SMD| = 0.1 and 0.2).
